# A study protocol for assessing the effects of intangible cultural heritage experiences on human well-being

**DOI:** 10.1371/journal.pone.0336120

**Published:** 2025-11-12

**Authors:** Alicia Núñez García, Sofia de la Fuente García, Erfan Lowemi, Masood Masoodian, Renata Vieira, Aurea Rodrigues, Saturnino Luz

**Affiliations:** 1 Usher Institute, Edinburgh Medical School, The University of Edinburgh, Edinburgh, Scotland, United Kingdom; 2 School of Arts, Design and Architecture, Aalto University, Espoo, Finland; 3 CIDEHUS - Centro Interdisciplinar de História, Culturas e Sociedades, Universidade de Évora, Évora, Portugal; PLOS: Public Library of Science, UNITED KINGDOM OF GREAT BRITAIN AND NORTHERN IRELAND

## Abstract

**Background:** While interventions have been designed which use extended reality (XR) technology in promoting physical, mental and social well-being through cultural heritage experiences, well-defined methodologies for the assessment of such interventions is lacking.

**Objectives:** We present a protocol for evaluating the usability and effectiveness of an XR system that mediates and facilitates access to intangible cultural heritage experiences. We aim to assess the effects of these experiences on user well-being and attitudes across four case studies: ageing societies, sustainable tourism, disappearing communities, and immigration and multiculturalism.

**Methods and analysis:** Participants will be randomly assigned to control or intervention groups. The effects of the XR intervention on well-being will be assessed through statistical analysis of the participants’ salivary cortisol and cortisone levels, physiological signals, and subjective ratings, both pre- and post-intervention and between control and intervention groups. Usability will be measured through a system usability scale. Speech will be recorded for qualitative and natural language processing analysis. Machine learning models will be developed for prediction of affect and well-being on multimodal data.

**Discussion:** This is one of the first international and multidisciplinary studies to explore the effects of XR-mediated intangible cultural heritage experiences on well-being and attitudes towards issues of societal importance. One of the main strengths of this study is the range of data modalities it collects, and the range of methods it employs to analyse these data in a complementary manner, including qualitative, statistical and advanced machine learning methods.

**Conclusion:** This protocol offers a method and four case studies to assess the potential of immersive XR experiences and interventions of intangible cultural heritage as contributors to increased well-being and as actors of societal change. It stands as a reference model for further similar interventions in the field.

## Introduction

Interest in the effects of cultural heritage experiences and human well-being has grown over the past decade, with studies identifying a range of impacts on everyday well-being in participants who have engaged with cultural heritage, including the strengthening of identity, psychological stability, self-esteem, capability, place attachment and sociability, among others [1–4]. Specifically, digital cultural heritage interventions that use virtual, augmented and extended reality and other interactive technologies to effect social change allow researchers to study experiential interactions with heritage in more accessible environments. This is an innovative strategy to engage and impact societies [5]. However, there is a pressing need to develop standardised assessment methods for digital cultural heritage [6].

In this context, we are investigating emotional, experiential and environmental dimensions of intangible cultural heritage in the Horizon Europe project INT-ACT (“Intangible Cultural Heritage, Bridging the Past, Present, and Future”). The project utilises XR technology to present these dimensions along with their associated tangible cultural heritage sites. It focuses on intangible cultural heritage—i.e., practices and aspects of culture that shape our understanding of ourselves, our sense of belonging, and our relationships to each other and to the environment—as a means of bridging the past, present and future to provide novel approaches to transforming society and addressing societal challenges [7]

According to the WHO, one in four people will experience a social or affective disorder globally throughout their lifetime [8]. Hence, research institutions worldwide are working towards new strategies to foster mental health and overall well-being. Recently, there has been a surge in the use of digital interventions for the promotion of health and well-being [9], especially in the wake of the COVID-19 [10]. We argue that assessing such approaches requires developing methods that combine multiple disciplines and evaluation approaches. The protocol described in this paper aims to contribute to this endeavour.

We draw on novel artificial intelligence methods, speech technology and multimodal signal processing, which have been increasingly researched as tools to monitor cognitive and mental health [11–15], to enable collection of behavioural data in natural settings for the purpose of evaluating XR-based cultural heritage experiences. Previous research has employed similar methods on naturally collected speech and behavioural data to assess cognition [16,17], emotional health [13,18] and general well-being [19]. Data collected through wearable digital devices can be harnessed to infer and promote well-being and mental health [9]. The proposed protocol combines these data with structured data collected through validated scales and questionnaires to enable a multi-faceted assessment of intangible cultural heritage experiences in our study.

The study selected four tangible cultural heritage sites as the basis for developing and creating content for four small-scale XR applications called “demonstrators”. These demonstrators are then used in four case studies dealing with cultural, social and technological changes facing citizens and cultural heritage, namely: cultural tourism, ageing societies, disappearing communities, and immigration and multiculturalism, with the overarching theme of well-being. The case studies are:

- Case Study 1 (CS1) - Cultural Tourism, Portugal. Focusing on the *Cromeleque das Fontainhas* (Mora, Alentejo), this case study will investigate ways of mitigating the potential negative impacts of cultural tourism, including overcrowding, environmental degradation, disruption to local populations, and cultural identity erosion [2,20] through the use of immersive XR environments. This can offer visitors a more engaging way to experience being at a cultural destination and interacting with its heritage without physically disturbing it.
- Case Study 2 (CS2) - Ageing Societies, Scotland. Focusing on the sites of *Calanais* (Isle of Lewis, Outer Hebrides), this case study will investigate the effects of intergenerational relationships on the well-being of older adults and healthy ageing [8]. Material collected as part of this study will be analysed to understand the ways in which well-being experiences regarding cultural heritage sites are described, and how their associated well-being experiences can be amplified and improved [3].
- Case Study 3 (CS3) - Disappearing Communities, Finland. Focusing on Koli National Park, this case study will identify the ways in which artists can help preserve the intangible cultural heritage of rural communities [21]. It will investigate how artists can take part in interpreting and re-inventing intangible cultural heritage, as well as extending its reach to the local populations and visitors alike. It will, therefore, investigate how the transformative human potential of art can be experienced in local intangible cultural heritage including folk arts and crafting skills, through creative community action that promotes emotional, experiential, and environmental resilience, and long-term sustainability [22–25].
- Case Study 4 (CS4) - Immigration and Multiculturalism, Greece. Focusing on the old quarter of Kavala, where emerging local populations (Greeks, Bulgarians and Turks) and migrants (southern Mediterranean and northern Europe) meet, this case study will trial the use of exhibitions, events, and educational programmes that highlight the cultural contributions, traditions and practices of different groups. It will create a space for dialogue and reflection on the challenges faced by immigrants and the local community, and how sharing cultural heritage can lead to better communication and understanding [26,27].

**Fig 1:**
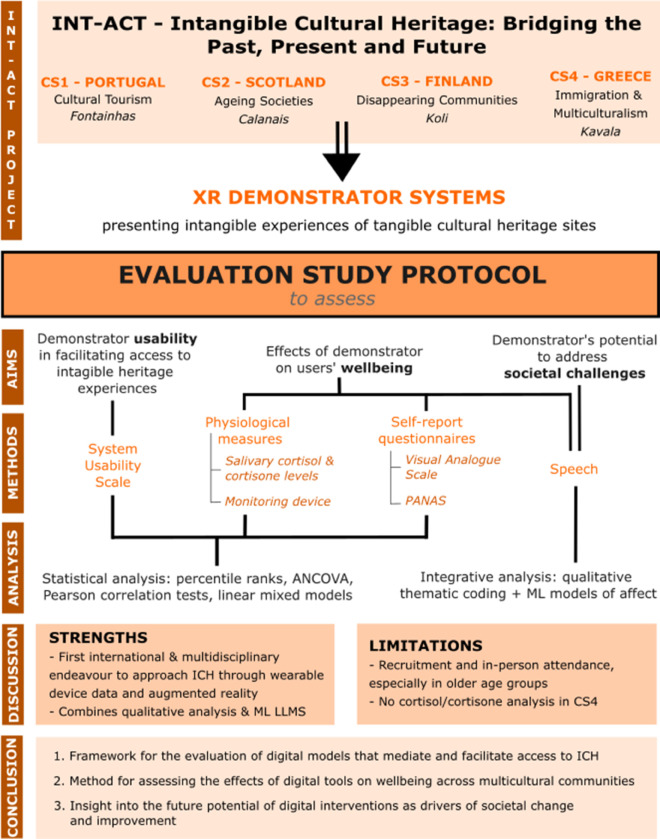
INT-ACT evaluation study protocol summary with a breakdown of aims, methodology and analysis.

This study protocol sets out the goals, procedures and methods for evaluating these four case studies (See Fig 1). The protocol aims to provide a method to test the feasibility of immersive XR-based intangible cultural heritage interventions as mediators and contributors to well-being, societal change and transformation.

## Materials and methods

### Objectives

This evaluation study has a threefold objective: (1) to evaluate the usability of a demonstrator XR system in providing access to intangible culture heritage experiences for people of different backgrounds, (2) to assess the effects of the demonstrator on its users’ general well-being, and (3) to assess its potential to address societal issues, including ageing societies, cultural tourism, disappearing communities, and immigration and multiculturalism.

Secondary objectives include (a) testing a machine learning (ML) and large language model (LLM)- based application that records and analyses the participants’ voice to determine affect, as a proxy for psychological well-being, and (b) connecting visitors with and improving their knowledge of the four chosen cultural heritage sites.

In terms of addressing societal issues (objective 3), we focus specifically on the issues of how to mitigate the potential negative impacts of cultural tourism, foster inter-generational relationships and belonging within an ageing population, promote the involvement of artists in interpreting and re-inventing intangible cultural heritage for the local populations and visitors alike, foster better communication and understanding between immigrants and local communities, in the context of cultural heritage sharing.

The case studies can be summarised in terms of the *PICO* framework [28], as shown in Table 1.

**Table 1:** Characterisation of studies in terms of the PICO framework.

Study	Population	Intervention	Comparators	Outcomes
Évora	local community	demonstrator 1	XR demonstrator vs booklet	Well-being, system usability, awareness of sustainable tourism
Calanais	older paired with younger adults	demonstrator 2	XR demonstrator vs video, pre/post intervention	Well-being, system usability, intergeneration engagement
Koli	artists, community	demonstrator 3	XR demonstrator vs regular visit	Well-being, usability, emotional, experiential and environmental resilience
Kavala	immigrant communities	demonstrator 4	XR demonstrator vs regular visit	Well-being, system usability, communication and understanding

This study was registered in the OSF Registries (Registration ID: 9gmpx), on May 28, 2025 [29], where a detailed account of study design, methodology and analysis plan can be found.

### Participant characteristics

Participants are healthy adult volunteers living in Portugal (CS1), Scotland (CS2), Finland (CS3) or Greece (CS4) at the time of study and able to give informed consent. For CS1, CS3 and CS4, participants will be over 18 years old at the time of initial consent. For CS2, participants should belong to the older range of population in Scotland, 60 years old or older at the time of initial consent, or, to a younger range, 18-30 years old, at the time of initial consent. Exclusion criteria for participants include suffering from severe hearing or speaking problems—i.e. incompatible with a spoken dialogue interview —, inability to speak fluent Portuguese (CS1), English (CS2), Finnish (CS3) or Greek (CS4), and lack of consent or ability to give it.

Participants will be identified according to age, locality and their involvement with cultural heritage, with support of local stakeholders, namely: Fundação Eugenio de Almeida and Mora Museum of Megalithism (Portugal), Historic Environment Scotland (HES), Koli Culture Society and Nature Centre Ukko (Finland), and Kavala Municipality (Greece). Participants will be informed of the study through a range of platforms including social media, newsletters, email lists, and media calls (e.g. radio), as well as individually. The recruitment period will run from July 2025 to June 2026, with workshops and open activities taking place between summer 2025 and summer 2026.

### Sample size

We aim to recruit around 200 participants in total - c.50 participants for each case study - who will be evaluated before and after being exposed to the XR demonstrators, therein conforming two groups for analysis, namely pre- and post- intervention. For well-being assessments, we will compare participants through their self-reports using the Positive and Negative Affect Schedule (PANAS [30]) scale, and a well-being visual analogue scale (VAS). We will also collect physiological measures of stress levels, namely, cortisol and cortisone levels through saliva swabs and physiological signals through wearable biometric devices. For self-reporting and VAS data, we estimate a sample size of 25 participants per group, based on a pre-post mean comparison, assuming a type I error probability α=0.05 and statistical power of 0.80, and aiming for a large effect size *d*>0.80, according to Cohen’s interpretations of effect sizes [31]. For physiological markers, we calculated the sample size following the analysis of a similar study [3]. In that study, differences in mean cortisol levels ($\bar{x}$) pre- and post-intervention ranged from ${\bar{x}}_{pre}-{\bar{x}}_{pos}=0.29-0.15=0.14\mu g/dl$ (morning) to 0.15−0.08=0.07μg/dl (afternoon), with an overall mean difference of 0.23−0.09=0.14μg/dl. The standard deviations (*s*) for the overall means were *s*_*pre*_ = 0.16 and *s*_*pos*_ = 0.07. This indicates a large effect size, $d=\frac{{\bar{x}}_{pre}-{\bar{x}}_{pos}}{\sqrt{(s_{pre}^{2}+s_{pos}^{2})/2}}=1.13$. The standard deviations for the afternoon readings (the worst case scenario) are 0.08 and 0.06, pre- and post-intervention respectively, resulting in a smaller effect size of *d* = 0.98. Based on this, we estimate a sample size between 35 and 46 participants to detect a statistically significant difference at the *p* < 0.05 level with power of 0.9. Overall, recruiting approximately 50 participants for each case study should provide the power we need for the intended statistical analyses. Power calculations for statistical analyses were performed with R, version 4.3.2 [32], using the *pwr* package [33].

For machine learning modelling, we aim to recruit the minimal number of participants required for learning algorithms to be able to generalise well-being and emotional factors (stress, anxiety, affect) in their predictions. While there is no clear consensus in the machine learning literature regarding sample size calculations, for relatively simple machine learning algorithms and metrics, such as Euclidean distance and linear discriminant analysis, lower bounds of 1.2 to 1.4 participants per feature (variable) in the model have been proposed, for an expected probability of misclassification at most 50% greater than an asymptotic value of 0.1 [34]. For estimating levels of emotional experience, if we take a previous study that employed the arousal-valence model as a guide [13], assuming a model R = 0.5, a regularisation factor of 0.8, mean arousal and valence scores of 30 (standard deviation of 22) and a reduced features set of 40 features, would require about 300 participants, according to the method proposed by Riley *et al*. [35]. As this calculation is rather conservative, and as we may be able to augment the data collected in this study with other data sets, if each site recruits 40-50 participants, we should still be able to build effective machine learning models.

### Machine learning methods

An effective strategy to address the relatively small size of the data set to be collected in this study is to leverage pre-trained models, which have been trained on large-scale, diverse data sets. These models, often based on architectures such as Transformers [36] (e.g., Whisper [37], BERT [38], and LLMs), capture rich, generalised representations of linguistic, acoustic, and contextual features. In the context of this study, these pre-trained models can be employed in a zero-shot or few-shot manner, directly applying their knowledge to the task or fine-tuning them on the smaller, domain-specific datasets collected within the project. Fine-tuning involves adapting the models to the unique linguistic, acoustic, and cultural characteristics of the INT-ACT data, thus improving their relevance and performance.

Additionally, transfer learning techniques such as domain adaptation and multi-task learning can be applied to make the models more robust to domain shifts and enhance their generalisability across related tasks, such as speech segmentation, diarisation, and sentiment analysis. Few-shot learning paradigms, supported by sophisticated data augmentation techniques like text paraphrasing, audio synthesis, or contrastive data sampling, can further mitigate the effects of limited training data. Finally, leveraging unsupervised or semi-supervised learning approaches, such as self-training or pseudo-labeling, can extract additional value from unlabeled or partially labeled data within the data set. By combining these strategies, we can significantly reduce the dependency on large volumes of labeled data while simultaneously enhancing the accuracy, robustness, and interpretability of the resulting models, ensuring their effectiveness in extracting meaningful insights from complex, interdisciplinary data sources.

### Study design

The study involves collecting and analysing recorded speech, well-being ratings, survey answers and saliva samples from participants collected over a two-day period - over three months for the whole evaluation. Participants will complete the study individually. For CS2, an older person (60 or older) will be paired with a younger person (30 or younger). Participants will be randomly assigned to a control group (CG) or an intervention group (IG). The control group will take part in a conventional cultural heritage activity related to each case study’s cultural heritage site, which will be supported by written material and visual resources available to the general public. The intervention group will explore the relevant intangible cultural heritage case study’s site using the INT-ACT XR demonstrator.

The following materials will be employed in the evaluation:

- A VAS, ranging between 0 and 100, to measure nervousness (nervous/calm), happiness (sad/happy), interest (bored/interested), and belonging (connected/isolated), as well as general well-being levels (wellness absent/maximum imaginable wellness). This has been adapted from Johnson *et al*.’s [39] VAS assessment of happiness, wellness, interestedness, confidence, and optimism in order to understand the effects of museum object handling and art viewing on early dementia and caregiver participants. It also encompasses Grossi *et al*.’s [3] single VAS of “wellness absent/maximum imaginable wellness” developed to measure the effect of cultural activities on stress reduction. This will allow subjective evaluation of the four pairs of co-related emotions informing the use and impact of the demonstrator— e.g. in fostering attention (bored/interested) and belonging (connected/isolated)—alongside a subjective assessment of general well-being.
- The PANAS scale, which consists of 20 items—i.e. words describing feelings and emotions —, scored in a 5-point Likert scale from "very slightly or not at all" to "extremely". Originally developed by Watson *et al*. [30], PANAS is a measure of positive and negative affect. It is brief and has been shown to be sensitive to short-term fluctuations, making it suitable for our two-day evaluation study. Besides, there is evidence of convergent validity between the Positive Affect subscale and social activity and diurnal variation in mood, as well as evidence of discriminant validity between the Positive Affect subscale and measures of stress, general distress and depression. The opposite is true for the Negative Affect subscales [40]. As a widely validated psychological scale with these characteristics, including PANAS serves a two-fold purpose. It provides a self-reported baseline in combination with the adapted VAS and contributes to its validation, while complimenting the robustness of the VAS and physiological measurements in evaluating the predictive ability of the machine learning models.
- A System Usability Scale (SUS), adapted to the INT-ACT demonstrator, as a measure of perceived system usability [41,42].

Each case study will be conducted according to the following procedure (Table 2). On Day 1, the activity will start with a familiarisation session (1 hour)—either individually or with an assigned pair (CS2)—to familiarise the participants with the demonstrator (in the case of the IG) or alternative material (CG). Additionally, study participants will be asked to complete the well-being VAS and PANAS, provide a saliva sample, and wear a physiological monitoring device [43] for the remainder of the study. This device will measure participant’s heart rate (HR), heart-rate variability (HRv), respiration, oxygen saturation, blood volume pulse (BVP), electrodermal activity (EDA), galvanic skin response (GSR), movement (acceleration, rotation, cardinal direction), skin temperature (SKT), for affect recognition.

**Table 2:** INT-ACT evaluation study design procedure.

	Session type	Session content	Duration
**DAY 1**	Familiarisation	Familiarise with demonstrator (IG) or alternative material (CG); VAS and PANAS; collection of saliva sample (same time as day 2); provided with physiological monitoring device	30min
**DAY 2**	Discussion	Discussion of study-relevant topics: sustainable tourism (CS1), ageing societies (CS2), disappearing communities (CS3), and immigration and multiculturalism (CS4); mood diary	30min
	Evaluation	Use of demonstrator (IG) or alternative material (CG)	1h
	Debrief	Conversation about the visit and impact on well-being; VAS and PANAS; SUS; collection of saliva sample (same time as day 1)	30min

On Day 2, prior to the activity, participants will be asked to discuss study-relevant topics (sustainable tourism, ageing societies, disappearing communities, and immigration and multiculturalism) according to each CS. These discussions will be audio recorded, using an encrypted audio recorder. A “mood diary”, where participants will be reflecting upon their general mood, will also be audio recorded. This will consist of spontaneous speech in response to the open question ‘how are you feeling?’.

The cultural heritage activity will be supported by the INT-ACT XR demonstrator (IG) or standard site material (CG). The participant (or pair of participants) will be given a single-user tablet to access the XR demonstrator (IG) or printed site materials (CG). Both groups will be provided with audio recorders and clip-on microphones, which they will use to record their impressions of their XR experiences.

Finally, a debriefing session will include a conversation about the visit, its impact on the participants’ well-being, their level of satisfaction with the activity and how aspects of intangible cultural heritage contributed to it, whether the demonstrator helped (or hindered) their engagement with the site, the perceived impact on their well-being, and any other insights relevant to the objectives of the study. This conversation will be audio recorded. Additionally, they will be asked to complete another well-being VAS and PANAS, provide a saliva sample following the above-described procedure, and assess the tool’s usability by completing the SUS.

#### Physiological samples.

The collection of saliva samples for measurement of salivary cortisol and cortisone levels directly relates to the secondary endpoint to rate the impact of the XR demonstrator on well-being. Low salivary cortisol levels are associated with positive affect [3,44], especially when combined with cortisone levels [45]. These will be used in this study to complement the well-being VAS and PANAS assessments. Saliva samples for the measurement of cortisol and cortisone levels will be collected using Salivettes^®^. Participants will be instructed on how to collect the sample and allowed to do it on their own, with a researcher available for questions or guidance as needed. Using a standard operating procedure, all researchers will direct participants to pour a swab from a tube directly into their mouth and roll it around for 1-2 minutes. After this time, participants will be asked to spit the swab back into the tube. A short leaflet guide for the participant (’How to take your saliva’) has been designed as support. Participants will be instructed in advance not to eat any major meal, smoke cigarettes, drink caffeinated beverages or fruit juices, or consume dairy products for at least one hour before providing their saliva samples.

### Data management and storage

Participation in this research study will be based on informed consent. The study will digitally collect the following types of data:

1. Full name/signature and email to consent
2. Age at the time of participation (but not date of birth)
3. Sociodemographic information: sex, gender, ethnicity, location, education, primary language; self-reported rating of mood. This will be fully anonymous data
4. Speech data, as part of the voice recordings of interviews, mood diaries and dialogues around the demonstrator’s use and impact

Name, signature, email, age, sociodemographic information and self-reported rating of mood (VAS and PANAS) will be collected digitally through LimeSurvey, an online survey server hosted at the University of Edinburgh. Audio-recordings will be collected in person by the INT-ACT project’s research teams in each country (Scotland, Portugal, Finland and Greece) using an encrypted recording device.

All participants will be assigned a unique identification code upon entering the study. This number will be used to identify the participant and thus personal details will not be held by members of our study.

Study data will be stored, accessed, and backed up according to a pre-defined common strategy following the INT-ACT project’s Data Management Plan [46]. During the study, data will be stored in encrypted and secure servers in line with the University of Edinburgh’s data protection policy, which abides by the General Data Protection Regulation (GDPR) and the Data Protection Act 2018.

#### Voice recordings, transcription and translation.

Pre-processing of voice recordings entails transcription and translation. Transcriptions will be done in-house by the Edinburgh research team using Whisper [37], an automatic speech recognition (ASR) model, along with Large Language Models (LLMs) such as LLaMa-3 [47], Mistral [48], and Gemma-2 [49] models, to process speech and language data. This involves working with four languages: English, Portuguese, Finnish and Greek. Translation into English, which is essential for our multilingual work, will be done using the Whisper model. LLMs excel at natural language processing tasks, helping to interpret complex information encoded in the text data.

While these models are designed to minimise biases through extensive training on diverse datasets, their use will be carefully assessed by the research team to ensure the fairness and accuracy of their output. Privacy is a key concern, especially in the case of cloud-based LLMs. In order to preserve participant privacy and comply with GDPR, no cloud-based models will be used in this study. All LLMs used in our data analysis will be hosted on local machines at the University of Edinburgh, ensuring that no participant data will be transmitted across the network. The models used in the study also comply with the University of Edinburgh’s data protection policies. All data transcripts will be pseudonymised by using identification codes, and no identifiable data or identifiable quotes will be used in the dissemination of the study findings.

Voice conversion will be employed to anonymise the audio recordings. Audio data and pseudonymised speech metadata—pause patterns, voice parameters, features sets, figures on turn-taking patterns—will be de-identified and stored indefinitely.

In all data coding and storage, we will use open, standard data formats so as to support interoperability.

#### Processing of saliva samples.

The saliva samples will be frozen at -20^°^C and stored until cortisol analyses are performed. Storage will follow each case study location, namely, Oral Biology and Salivary Proteomics Laboratory of the Mediterranean Institute for Agriculture, Environment and Development (MED), University of Évora (CS1); The Clinical Research Facility, at the University of Edinburgh (CS2); Nanomicroscopy Centre (OtaNano), at Aalto University (CS3). CS4 will not include cortisol analysis due to a lack of facilities and tighter bio-sampling concerns due to migration in the locality.

Cortisol and cortisone analysis will be sought with The University of Edinburgh’s Clinical Research Facility at the Edinburgh BioQuarter, following procedures outlined by Gregory *et al* [45]. Samples will be shipped to Edinburgh from Portugal (CS1), and Finland (CS3) by courier service. Samples will be shipped in tubes, vertically placed in a tight box with dry ice and instructions for the courier to repack the ice every 24 hours if there are delays.

### Ethical considerations, declarations

This study has been granted ethical approval by the Edinburgh Medical School Research Ethics Committee (25-EMREC-004, 31 March 2025; and later with approved amendments 25-EMREC-004-A01, 19 May 2025) and complies with advice from its academic sponsor, the Academic and Clinical Central Office for Research and Development (ACCORD), at The University of Edinburgh. There are no expected risks to participants, who are healthy consenting adults informed that this is an non-invasive, exploratory study with no diagnostic aim.

Participants are free to withdraw from all aspects of the study at any point without providing a reason. The project will retain the right of continued use of the data collected up to that point. To safeguard rights, the minimum personally-identifiable information possible will be collected.

Data protection procedures comply with the requirements of the sponsoring institutions’ ethics committees, the EU’s GDPR and the UK’s Data Protection Act, 2018. In compliance with ethic guidelines and open science initiatives, the study was registered with OSF Registries (Registration ID: 9gmpx) [29]. The main ethical consideration for this study relates to data confidentiality, as it involves collection of audio data, deemed to be identifiable. Voice conversion will be employed to anonymise the audio recordings. Science and public communications will only include results on analyses undertaken after pre-processing the voice recordings and well-being survey data, ensuring that audio data or any other identifiable information will never be published or disseminated.

## Analysis

Survey responses on societal issues, baseline well-being self-report responses and usability questionnaire data will be analysed though descriptive and inferential statistics. The study has a mixed design, with a *within-subjects* component, which compares the same participants at two different time points, namely, before and after exposure to the cultural heritage intervention (pre- and post-, repeated measures); and a *between-subjects* component, which compares participants in the control group (exposure to standard site materials) with participants in the intervention group (exposure to XR demonstrator) at a given time point.

### Statistical analysis

- **Baseline well-being index responses**. Well-being VAS, PANAS and cortisol levels pre- and post-intervention and between CG and IG will be compared through ANCOVA, while correlations between VAS, PANAS and cortisol analyses will be drawn through Pearson correlation tests. Generalised linear mixed models will also be used as appropriate after data exploration.
- **ML algorithms** will be employed to create models of affect and well-being against a VAS target function, using speech and wearable device data. The predictive accuracy of these models will be assessed in terms of correlation coefficients and root mean square error measures.
- **SUS scores** will be normalised to a percentile rank for better visualisation and compared among case studies. The statistical inference techniques mentioned above will also be used to approach SUS analyses as appropriate.

### Integrative analyses

The study will integrate qualitative analysis with ML and LLMs. Thematic analysis through deductive manual coding will explore themes related to past, present and future, memory, and perspectives pertaining to each case study. Focus will be given to:

- CS1. Potential negative impacts of cultural tourism and alternative solutions through sustainable cultural tourism practice
- CS2. Comparison of improvement in mental well-being for ageing participants and intergenerational aspects
- CS3. Ways in which artists can help to combat the disappearance of rural communities and cultures
- CS4. Comparison of improvement in local-immigrant connection and other aspects of multiculturalism

This will be integrated and complemented with LLMs “theme and sentiment” analyses which can identify both explicit and nuanced themes, analyse sentiment, and provide structured insights from textual data. By generating concise summaries, extracting keywords, and scoring themes based on their relevance, LLMs can significantly enhance the depth and efficiency of the analysis. However, challenges such as bias in the training data of LLMs can influence the accuracy and objectivity of the results. These biases, if not carefully managed, can perpetuate stereotypes or misrepresent certain narratives, potentially impacting the integrity of the findings. Addressing such challenges requires careful evaluation, fine-tuning, and validation of the models to ensure their outputs align with the goals of the study.

## Discussion

A key strength of the study protocol described in this paper is the broad range of contexts, modalities and analytical methods employed. To the best of our knowledge, this is the first international and multidisciplinary endeavour to approach intangible cultural heritage through wearable device data and augmented reality, combined with qualitative analysis. The study design balances control (i.e. allocation to group conditions) and naturalness (i.e. focus on participant’s immersive experiences and spontaneous interaction with the environment), which enhances the external validity of the study. Methodologically, the study will tackle the challenge of combining qualitative analysis with machine learning and LLMs. We aim to present the wider research community with a thorough discussion on this challenge and the lessons learned in the project.

Beyond its academic contributions, the findings of this evaluation protocol are expected to have practical implications for cultural institutions, policy-makers, and the XR industry. By systematically assessing the impact of XR-mediated intangible cultural heritage experiences on well-being, the study offers evidence-based insights that can inform the design of future digital heritage interventions in museums and heritage sites. Furthermore, the evaluation framework may support cultural policy initiatives aiming to promote inclusivity, digital accessibility, and social cohesion through heritage. The study’s outcomes can also guide developers and curators in creating more user-centered XR experiences that are both meaningful and culturally sensitive.

Results of the evaluation will be published in peer-reviewed journals, aiming for an interdisciplinary audience and with a focus on digital cultural heritage and its relation to well-being, as well as themes arising from each of the specific case studies. The authors foresee potential use of the demonstrator and this study evaluation in future projects or redevelopment into future versions of the programme.

Potential limitations of this study design include difficulties in recruitment and attrition, as participants will be required to attend the study in person, which may restrict participation, especially among older participants. In order to mitigate recruitment and retention risks, we will undertake focused recruitment efforts to reach the groups that we think will be harder to recruit, such as older adults. Furthermore, in CS4, there will not be a cortisol/cortisone analysis so as to allay potential misgivings from migrant communities concerning biometric surveillance.

## Conclusion

The potential of digital experiences and interventions of intangible cultural heritage as promoters of well-being and as vehicles of societal change is a promising research field. The protocol presented in this paper offers (1) a framework for the evaluation of digital models that mediate and facilitate access to intangible cultural heritage, (2) a method for assessing the effects of the INT-ACT demonstrator, and similar digital tools, on well-being across four multicultural communities and (3) an insight into the future potential of such digital interventions as drivers of societal change and improvement, relating to issues of ageing societies, sustainable tourism, disappearing communities, and immigration and multiculturalism.

## Abbreviations

ASR: Automatic speech recognition
CS1-4: Case studies 1-4
CG: Control group
IG: Intervention group
INT-ACT: Intangible Cultural Heritage, Bridging the Past, Present and Future
LLM: Large language model
ML: Machine learning
PANAS: Positive and Negative Affect Scale
SUS: System Usability Scale
VAS: Visual Analogue Scale
WHO: World Health Organisation
XR: Extended reality
